# Systematic realization of double-zero-index phononic crystals with hard inclusions

**DOI:** 10.1038/s41598-018-25696-y

**Published:** 2018-05-08

**Authors:** Jaeyub Hyun, Wonjae Choi, Semyung Wang, Choon-su Park, Miso Kim

**Affiliations:** 1 School of Mechatronics, Gwangju Institute of Science and Technology (GIST), 123 Cheomdan-gwagiro, Buk-gu, Gwangju 61005 Republic of Korea; 2 Center for Safety Measurement, Korea Research Institute of Standards and Science (KRISS), 267 Gajeong-ro, Yuseong-gu Daejeon, 34113 Republic of Korea; 3 Present Address: Center for Medical Metrology, Korea Research Institute of Standards and Science (KRISS), 267 Gajeong-ro, Yuseong-gu Daejeon, 34113 Republic of Korea

## Abstract

A systematic process is described to realize double-zero-index phononic crystals with Dirac-like points experimentally. This type of crystal normally has softer inclusion material than its surroundings medium, allowing mapping into a zero-index medium under certain conditions but also making experimental implementation difficult. On the other hand, realizing phononic crystals with hard inclusions can be experimentally more feasible, but the mapping conditions cannot be directly applied to hard-inclusion crystals such that mapping is not systematically guaranteed in these cases. Moreover, even if such crystals become realizable, there is a lack of a systematic design process which can be used to optimize or to redesign the crystals, which largely limits their potential applications. In this paper, we discover the essential conditions for realizing phononic crystals with hard inclusions and propose a methodology for the systematic design of these crystals using homogenization based on the effective medium theory. Using the proposed method, a double-zero-index phononic crystal with hard inclusions is optimized and experimentally realized for an underwater ultrasonic wave collimator.

## Introduction

Metamaterials have been widely investigated in recent years. These are artificially designed to realize unprecedented physical characteristics such as negative refraction and bandgap^1,2^. A zero-index metamaterial is one of the types of materials which exhibit zero refractive indices, and these materials can be realized by several phenomena, including local resonance^3^, zeroth-order Fabry-Perot resonance^4^, and with a Dirac-like point^5^. We focus here on the last method, particularly for underwater applications. The first realization of a zero-index medium based on a Dirac-like point was achieved in the optic/electromagnetic field in 2011^6^, and an equivalent phononic crystal was theoretically realized in 2012 through an acoustic analogy to the phenomenon of an optic/electromagnetic Dirac-like point^7^. At a Dirac-like point with a properly designed crystal, both the effective density and the reciprocal of the effective bulk modulus can be zero, resulting in double-zero-index characteristics. The crystal shows better transmission performance due to its finite acoustic impedance^8^ than a single zero-index material with a near-infinite impedance mismatch, leading to many interesting applications such as unidirectional transmission^9^, cloaking^10^ and tunneling effects^11^ with little transmission loss.

Recent studies^6–8,12^ have shown that crystals with a Dirac-like point can be mapped to a double-zero-index medium (double-ZIM) *only if* the Dirac-like point firstly originates due to a linear combination of monopole and dipole modes and secondly is formed at or near a Brillouin zone (BZ) center or ${\rm{\Gamma }}$ point. These two conditions were found for photonic crystals having *soft* inclusions surrounded by a hard matrix^6^. Analogues to the photonic crystal, double-zero-index phononic crystals (DZIPnCs), are often designed with *soft* inclusions in hard-matrix materials or with slow inclusions in a fast matrix, such as air cylinders in a water tank^13^, rubber cylinders in water^7^ or rubber cylinders in an epoxy host^14^. Previous efforts demonstrated the possibility of phononic crystals using numerical simulations, but DZIPnCs with such *soft* inclusions are difficult to realize experimentally because the matrix is normally the slowest or softest material, such as air and/or water. Although one study^8^ described an experimental observation of a phononic crystal exhibiting a near-zero feature in an air medium, the inclusions were still softer than the matrix. If DZIPnCs are achieved with acoustically *hard* inclusions, they can be readily implemented in various applications. Recently, an example of a zero-index phononic crystal with *hard* inclusions was simulated with a Dirac-like point at the BZ corner or M point^15^. It is interesting that the crystal still appears to have double-zero-index attributes, despite the fact that the Dirac-like point at the BZ corner violates the second mapping condition mentioned above. The mapping conditions for such crystals are not yet known though they are unveiled in this paper.

In addition, the lack of systematic process in the design of acoustic metamaterials and phononic crystals is another bottleneck preventing their realization in industrial applications. Research is often based on trial-and-error methods and intuitive approaches; thus, optimizing the crystals or redesigning them for other applications becomes extremely laborious and time-consuming. For instance, in order to confirm the presence of a ZIM property, one can analyze acoustic wavefronts after a waveguide of an array of crystals. Such an indirect method requires a large amount of computation labor and does not provide physical insight into the principle of DZIPnCs. Thus, in order to improve the computation efficiency in this case, several inverse-design cases based on optimization algorithms have been attempted recently^16–18^. To the best of our knowledge, however, an inverse design of a DZIPnC has not been reported. Meanwhile, it is necessary to define quantitative parameters for an inverse-design method, and the effective medium theory (EMT)^19^ has been widely used to provide these parameters^20^. According to this theory, the effective density and effective bulk modulus are calculated and represent the local material properties of the DZIPnC. The EMT within a unit cell is known to be applied in the immediate vicinity of the BZ center in general; otherwise, the quality of the calculated properties cannot be guaranteed^19,21,22^. Therefore, it is important to ensure that the Dirac-like point is located at the BZ center in order to apply the EMT within a unit cell.

In this paper, in an effort to realize a double-zero-index phononic crystal with *hard* inclusions systematically, we discover the general conditions required to map phononic crystals with *hard* inclusions at a Dirac-like point onto a zero-index medium and propose a systematic inverse-design methodology for designing DZIPnCs. Before introducing the inverse-design method, we explicate the mapping conditions by comparing them with those of crystals with *soft* inclusions. A zone-folding mechanism is then introduced to locate the Dirac-like point at the BZ center so as to apply the effective medium theory accurately. An inverse-design method with bi-objective functions is described to design the DZIPnC with a Dirac-like point systematically. Finally, experimental realization of the optimized DZIPnC is achieved with copper inclusions and a water matrix in the ultrasonic regime for underwater applications.

## Results

### Zero-index mapping of a hard-inclusion crystal with C_4v_ symmetry

As discussed in the previous section, the mapping conditions for a DZIPnC with a Dirac-like point require the Dirac-like point to be at the BZ center (i.e., the ${\rm{\Gamma }}$ point) and constructed via a linear combination of a monopole mode and dipole modes. However, these apply to photonic crystals with *soft* inclusions and not to phononic crystals with *hard* inclusions and thus cannot be directly applied to phononic crystals. In this section, we briefly explain the mapping conditions for photonic crystals and describe the difference between cases with *soft* and *hard* inclusions, after which we unveil the mapping conditions for *hard*-inclusion phononic crystals.

The conditions can be explained through the spatial symmetry characteristic of Bloch modes. In particular, the relationship between a combination of several Bloch modes and the construction of a Dirac-like point had been comprehensively analyzed in terms of this type of mode symmetry in previous studies^23–25^. For square lattice crystals with ${{\rm{C}}}_{4{\rm{\nu }}}$ symmetry (i.e., where the symmetry is preserved upon a rotation of 90° (=360/4)), combinations of Bloch modes at the Dirac-like point at the BZ center (i.e., ${\rm{\Gamma }}$ point) are categorized into two types for *soft* inclusions, as shown in Table 1: (1) a monopole mode and dipole modes, and (2) a quadrupole mode and dipole modes^24^. However, not all of these mode combinations can be mapped to a double-ZIM. Because the surrounding medium is acoustically harder than the inclusions, the acoustic energy is localized at the inclusion area near the center of the unit cell. Thus, the monopole mode is readily excited at a relatively low frequency compared to the quadrupole mode, and the first combination (i.e., a monopole mode + dipole modes) in Table 1 can be mapped to a ZIM. With the other combination of a quadrupole mode and dipole modes listed in the table, the crystal cannot easily be mapped to a ZIM^23^, as a higher operational frequency may be required. Alternatively, a core-shell structure may be utilized. Therefore, the first combination is the only possible mapping condition for a *soft*-inclusion crystal.

**Table 1:** Zero-index mapping condition for a square crystal with C_4v_ symmetry.

Inclusion-type	Position of a Dirac-like point within an irreducible BZ	Combination of Bloch modes to generate a Dirac-like point^24,26^	Mapping to the double-zero-index medium
*Soft* inclusion	BZ center (${\rm{\Gamma }}$ point)	Monopole (${{\rm{A}}}_{1}$) + Dipoles (${\rm{E}}$)	Yes
		Quadrupole (${{\rm{B}}}_{1}{\text{or}B}_{2}$) + Dipoles (${\rm{E}}$)	No
*Hard* inclusion	BZ corner (M point)	Monopole (${{\rm{A}}}_{1}$) + Dipoles (${\rm{E}}$)	No
		Quadrupole (${{\rm{B}}}_{1}{\text{or}B}_{2}$) + Dipoles (${\rm{E}}$)	Yes

An analogous condition can be found for *hard*-inclusion crystals, but it requires a relaxed condition of the BZ center. It is important to note that the M point has ${{\rm{C}}}_{4{\rm{\nu }}}$ symmetry identically to the ${\rm{\Gamma }}$ point within the BZ of the square crystal^26^. Given the identical symmetry characteristics of ${\rm{\Gamma }}$ and M points, it can be expected that the two combinations for *soft* inclusions can be directly applied to cases of a Dirac-like point at an M point. In such crystals, the acoustic energy is confined to the surrounding *soft* medium; thus, a quadrupole mode is readily excited, in contrast to *soft*-inclusion crystals. Therefore, by comparing *soft* and *hard* inclusion cases, crystals with *hard* inclusions can be mapped to a zero-index medium *only if* the Dirac-like point originates from a linear combination of a quadrupole mode and dipole modes and is located at a BZ corner. Table 1 summarizes these mapping conditions.

In this section, we generalize the mapping conditions of DZIPnCs with *soft*/*hard* inclusions. Interestingly, the combination of these Bloch modes (i.e., a quadrupole mode and dipole modes) for *hard*-inclusion crystals appears to violate the aforementioned conventional mapping conditions of a DZIPnC with *soft* inclusions, but the difference can be explained by the symmetry between *soft* and *hard* inclusions. Although this study uses phononic crystals with ${{\rm{C}}}_{4{\rm{\nu }}}$ symmetry as an example, an identical description can be extended to photonic crystals for both optics and electromagnetics with other well-defined instances of symmetry.

### Zone-folding for the Dirac-like point at k = 0

The EMT has been widely used to find quantitative material properties in the inverse-design process. In general, the EMT works well with the long-wavelength limit assumption of the unit cell^19,27^; therefore, in order to ensure the accuracy of the EMT, the location of the current Dirac-like point must be near the BZ center (i.e., the ${\rm{\Gamma }}$ point). Because the Dirac-like point of the DZIPnC of interest is located at the M point, we propose a zone-folding mechanism (i.e., enlarging the size of the unit cell to be analyzed) in order to move the Dirac-like point from the M to the ${\rm{\Gamma }}$ point.

**Figure 1:**
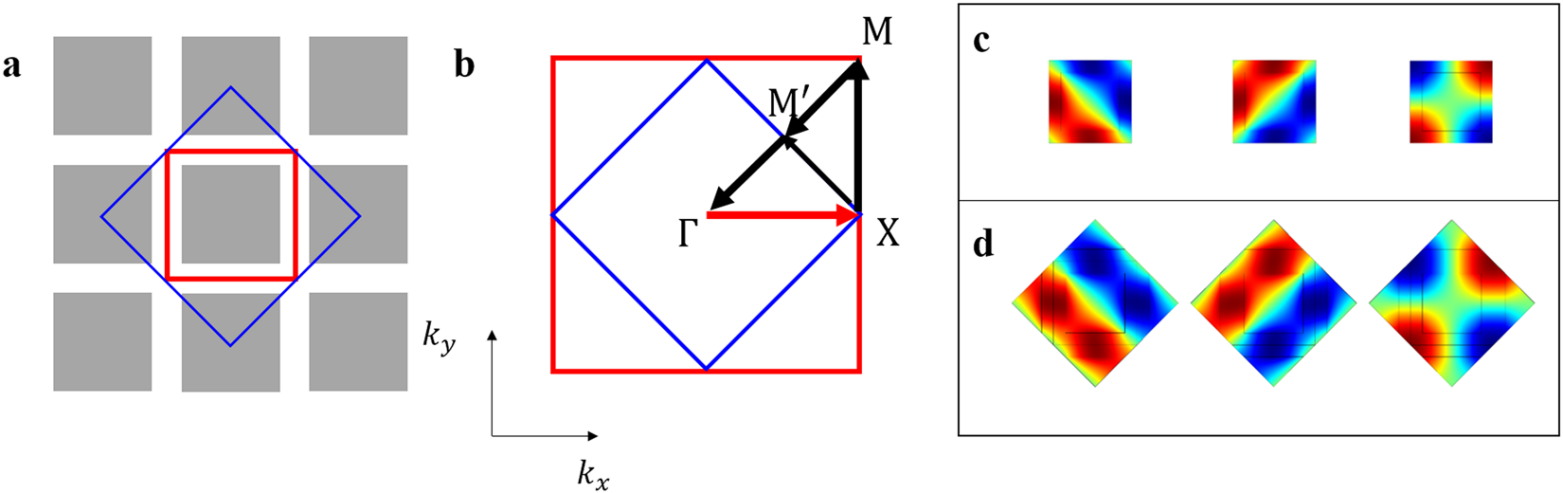
Two types of unit cells considered and their modes: (**a**) Original (O) and enlarged (E) unit cells. The red solid square represents unit cell type O in a conventional manner and blue solid diamond is unit cell type E. **(b)** The first Brillouin zones for two types of unit cells. The blue solid diamond denotes unit cell type E and the red solid square represents the zone for unit cell type O. (**c**,**d)** Bloch modes of unit cells at a Dirac-like point. Both unit cell types O and E have one quadrupole mode and two dipole modes.

**Figure 2:**
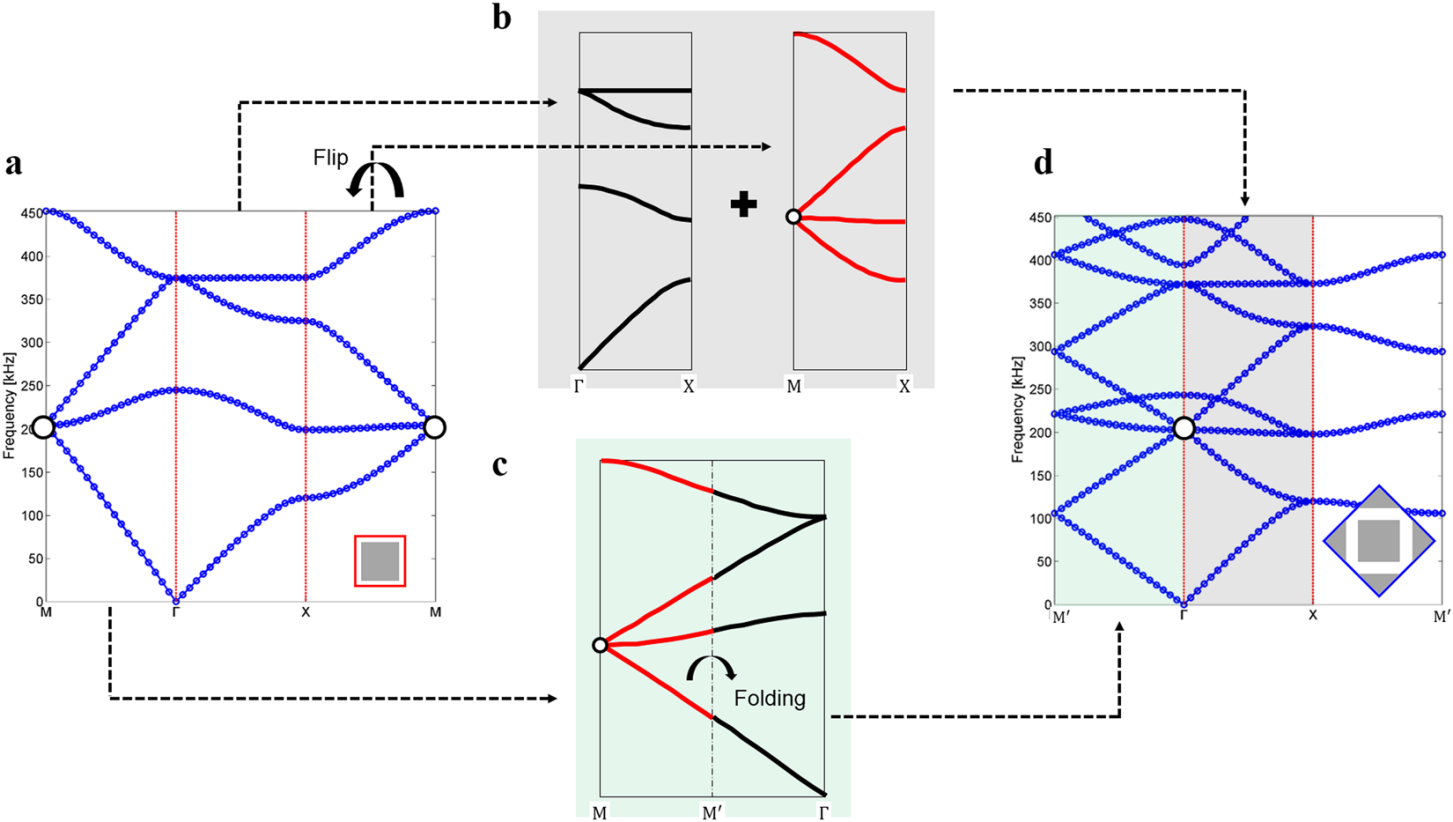
Band structures for the three types of unit cells and related folding mechanisms: (**a)** Band structure of unit cell type O and **(b**,**c)** zone-folding mechanism when the unit cell is enlarged. **(d)** Band structure of unit cell type E.

We selected a fully viable design of a DZIPnC as in Fig. 1, which already has a Dirac-like point^15^, in order to show that this zone-folding method is an essential foundation for the subsequent inverse-design process of the DZIPnC. From the band structure of the original unit cell in Fig. 2a, it is apparent that the Dirac-like point will be at the ${\rm{\Gamma }}$ point if the band structure on ${\rm{\Gamma }}\to {\rm{M}}$ is folded. One method which can be used for this purpose is to enlarge the size of the unit cell. When the area of the original unit cell (i.e., the red solid square in Fig. 1a) is doubled in size, as in Fig. 1a, its BZ in the reciprocal lattice is reduced to half (Fig. 1b). The band structure of the enlarged unit cell then becomes more complicated than the original case as a result of the zone-folding mechanism. Based on the notation shown in Fig. 1b, the band structure of the enlarged unit cell on $X\to {\rm{\Gamma }}$ will include the original structure for ${\rm{X}}\to {\rm{\Gamma }}$ as well as ${\rm{M}}\to {\rm{X}}$ due to the zone-folding mechanism. In addition, ${\rm{\Gamma }}\to {{\rm{M}}}^{\text{'}}$ can be constructed by folding ${\rm{\Gamma }}\to {\rm{M}}$ in the figure. These folding processes in the band structure can be seen in Fig. 2b,c, respectively. After the zone is folded by enlarging the size of the unit cell, the Dirac-like point is then located at the ${\rm{\Gamma }}$ point, as shown in Fig. 2d. Bloch modes of the enlarged unit cell are shown in Fig. 1d; these are still one quadrupole mode and two dipole modes.

The zone-folding mechanism makes the wavelength at the Dirac-like point relatively large enough to compute the effective medium within a cell, under the long-wavelength assumption in EMT. By moving the M point to the ${\rm{\Gamma }}$ point, the smallest wavelength of the unit cell can effectively become the largest one; thus, the crystal has the isotropic characteristic of an effective medium (See Supplementary Note for details) by augmenting the size of the unit cell. It is interesting to note that the periodic arrays of the original unit cell and of the enlarged unit cell are identical to each other despite the fact that the related band structures are different, as shown in Fig. 2a and d. By only changing the means of interpretation, the EMT process becomes extremely efficient because the homogenization step can be executed at the unit-cell level, which is normally much smaller than the waveguide. Moreover, this folding mechanism can be applied when designing any unit-cell-based photonic and phononic crystals.

### Systematic inverse design of the zero-index phononic crystal by optimizing the effective material properties and Dirac-like point

In this section, a systematic inverse-design method for DZIPnCs is proposed by combining the proposed unit-cell analysis method based on zone folding and the optimization method. The proposed inverse-design method can be applied to *hard* inclusions as well as to *soft* inclusions, but this study focuses particularly on a DZIPnC with a *hard* inclusion. First, for the inverse design of the DZIPnC, it is most important to define the optimization formulation with a physically quantifiable objective function. Physically, the DZIPnC should exhibit both near-zero properties and a Dirac-like point at the ${\rm{\Gamma }}$ point. Thus, the objective function $(J)$ can be defined as a bi-objective function that combines two sub-objective functions $({J}_{{\rm{H}}}\,\& \,{J}_{{\rm{D}}})$ based on a weighted-summation approach^28^. The two sub-objective functions must (1) make the effective properties, such as effective mass density and effective reciprocal of the bulk modulus, close to zero (${J}_{{\rm{H}}}$), and (2) make the Dirac-like point occur at the ${\rm{\Gamma }}$ point ($\,{J}_{{\rm{D}}}$).

1 $$J={W}_{{\rm{H}}}{J}_{{\rm{H}}}+{W}_{{\rm{D}}}\,{J}_{{\rm{D}}}$$

Here, the subscripts H and D denote ‘for homogenization’ and ‘for a Dirac-like point’, respectively. In Eq. (1), appropriate weighting factors *W*_D_ and *W*_H_ for each sub-objective function should be selected. Each weighting factor is automatically updated from the current values of the objective functions during the optimization process, as follows:

2 $${W}_{{\rm{H}}}=\frac{{J}_{{\rm{D}}}}{{J}_{{\rm{D}}}+{J}_{{\rm{H}}}}\,{\rm{and}}\,{W}_{{\rm{D}}}=\frac{{J}_{{\rm{H}}}}{{J}_{{\rm{D}}}+{J}_{{\rm{H}}}}.$$

The first sub-objective function ${J}_{{\rm{H}}}$ can be expressed as

3 $${J}_{{\rm{H}}}={(\frac{{\rm{R}}[\bar{\rho }]}{{\rho }_{w}})}^{2}+{(\frac{{\kappa }_{w}}{{\rm{R}}[\bar{\kappa }]})}^{2},$$

where R[] denotes ‘real component of’ and the density and bulk modulus of water are ${\rho }_{w}$ and ${\kappa }_{w}$, respectively. The equation above requires the effect properties, which can be calculated as shown below.

4 $$\bar{\rho }=\frac{\langle \nabla p\rangle }{j\omega \langle v\rangle }\,{\rm{and}}\,\bar{\kappa }=\frac{j\omega \langle p\rangle }{\langle \nabla \cdot v\rangle }$$

Here, $\langle \cdot \rangle$ refers to the average over the boundaries, *j* is the imaginary unit ${j}^{2}=-\,1$, $\omega$ is the radian frequency, and *p* and *v* are the pressure and particle velocity, respectively (for the average calculations, refer Supplementary Note). In order to create a convex-type function which improves the convergence performance of the optimal solution, squares were used for both normalized effective material properties, as presented in Eq. (3). Although the ideal conditions for the DZIPnC would be $\bar{\rho }=1/\bar{\kappa }=0$, we determine the two effective properties of the DZIPnC in Eq. (4) to make them very small relative to those of the surrounding medium, as in Eq. (3), in practice. The second sub-objective function ${J}_{{\rm{D}}}$ is to minimize the difference between the target operational frequency and the frequencies computed for each branch at the Dirac-like point,

5 $${J}_{{\rm{D}}}=\sum _{i=k}^{k+2}{({f}_{i}^{{\rm{\Gamma }}}-{f}_{0})}^{2},$$

where ${f}_{i}^{{\rm{\Gamma }}}$ is the frequency computed for the *i*-th branch at the Γ-point, ${f}_{0}$ is the target operational frequency, and the *k* is the selected branch number. Through this optimization formulation with the physically quantifiable objective function, we developed an in-house program to perform the inverse design of the DZIPnC based on a genetic algorithm (GA).

**Figure 3:**
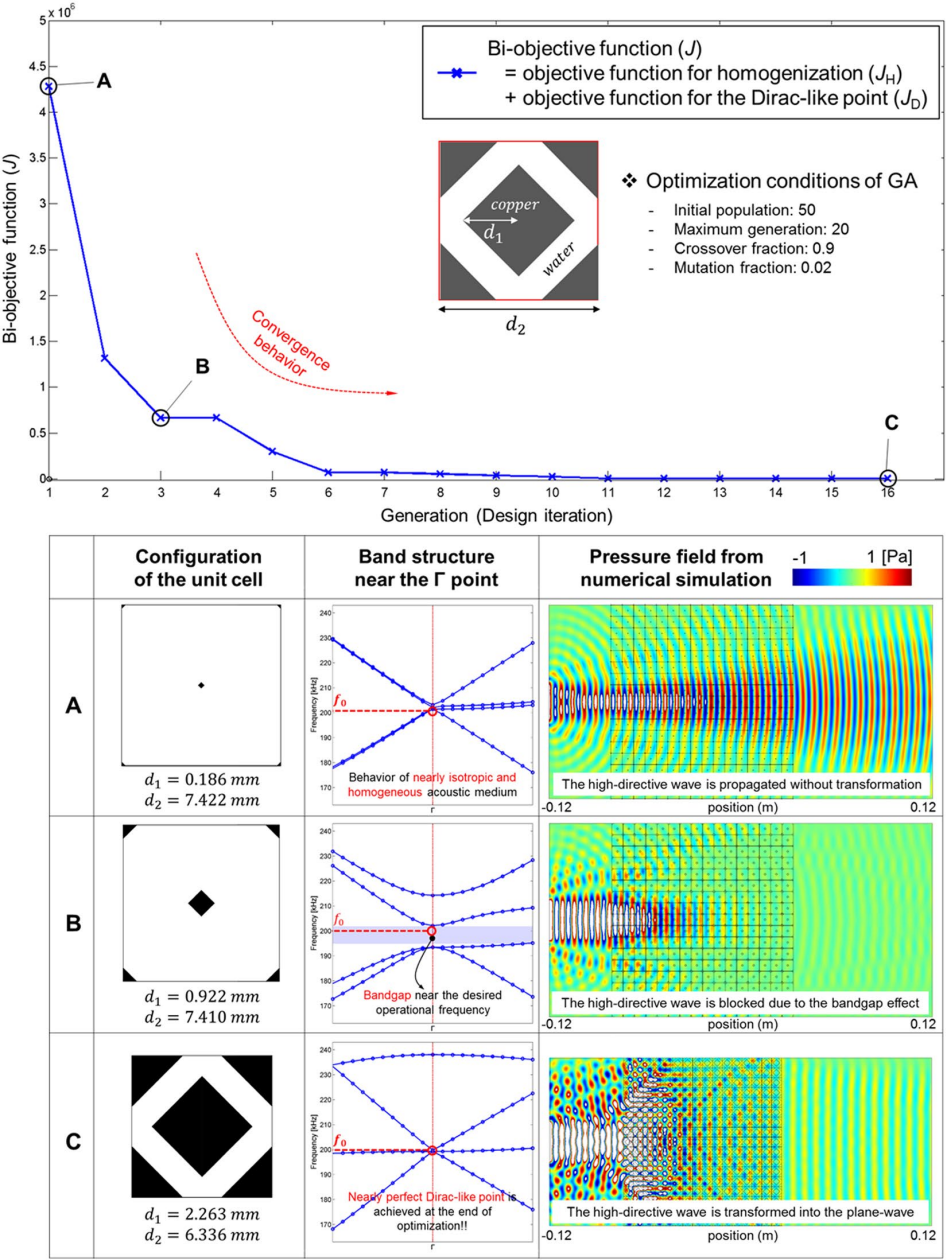
Optimization history of the DZIPnC with a square-shaped hard inclusion. Upper panel: the bi-objective value in an optimization history plot with respect to the design iterations. The inset shows the target unit-cell shape parameterized by two design variables. Lower panel: results at three iteration points in the optimization history. The figure shows the configurations of the unit cell (left), the band structures near the ${\rm{\Gamma }}$ point (middle), and the simulated pressure fields (right). The evolution of the configuration of the unit cell is evident and the configuration in step C is the unit-cell shape of the DZIPnC optimized from the GA algorithm.

In order to describe the inverse-design process, we present an example case of a DZIPnC for the design conditions specified in Table 2. Note that for an intuitive implementation during the process, the direction of the incident acoustic wave is aligned to the principal axis (e.g., the ${\rm{x}}$-direction); thus, we rotate the original enlarged unit cell shown in Fig. 1d by 45°. The rotated and enlarged unit cell is used as the basic crystal structure for the inverse design of the DZIPnC. A unit cell with two simple geometrical design variables (i.e., *d*_1_ and *d*_2_) is considered as the target crystal structure, as shown in the inset in the upper panel in Fig. 3. Here, *d*_1_ and *d*_2_ represent the size of the inclusion and that of the unit cell, respectively. The optimization history is presented in Fig. 3. The GA internally has two mathematical operators: crossover and mutation^29^. The controllable fraction parameters of these operators should be appropriately selected, and they are set to 0.9 and 0.02, respectively, here. In addition, in order to obtain a solution close to the global optimum, the size of the initial population and the number of maximum generation instances are set to 50 and 20, respectively. In addition, *k* in Eq. (5) is set to 2 in this example, as the target bands are from the second to the fourth branch. In the initial stage of the optimization process (step A), the propagation characteristic of the acoustic wave is mostly unaffected by the crystals. As the optimization progresses, the two design variables are gradually updated, and a bandgap starts to appear at the BZ center (step B). In the final stage (step C), both the Dirac-like point and the near-zero index feature are obtained; therefore, an almost perfect plane wave is achieved by the optimization process. The design variables *d*_1_ and *d*_2_ are found to be 2.263 mm and 6.336 mm, respectively, and the phononic crystal acts as a zero-index material at approximately 200 kHz. Using the EMT, we found the equivalent material properties to be ${\rm{Re}}[\bar{\rho }]/{\rho }_{w}=0.0515$ and ${\kappa }_{w}/{\rm{Re}}[\bar{\kappa }]=0.0371$. A geometrical sensitivity analysis was also conducted according to changes in the two design variables, *d*_1_ and *d*_2_. The near-zero-index feature (i.e., minimizing the phase change through the waveguide) was found to be guaranteed in the range of an approximately ±10% change of *d*_1_, although the amplitude of the transmitted wave decreases (for more details, refer to the Supplementary Note).

**Table 2:** Specified design conditions for a DZIPnC with a hard inclusion.

Target operational frequency (kHz)	Material of inclusion	Shape of inclusion
200	Copper	Square

Note that the inverse-design methodology is versatile when designing the DZIPnC and can be used to define a variety of design parameters, such as the material properties and dimensions of the unit cell as well as its inclusions. In addition to the example presented here, a DZIPnC with a circular inclusion was optimized at approximately 50 kHz. These results are shown in the Supplementary Note. It is also important to note that the inverse design of the DZIPnC is conducted through a single unit cell of the DZIPnC and not through the entire waveguide system composed of arrays of unit cells. Therefore, the speed of the analysis and design can be dramatically accelerated. If the aforementioned conditions (i.e., the Dirac-like point and the near-zero effective properties) are satisfied, the proposed GA-based inverse-design method can be applied not only to a square crystal with ${{\rm{C}}}_{4{\rm{\nu }}}$ symmetry but also to a triangular crystal with ${{\rm{C}}}_{6{\rm{\nu }}}$ symmetry. In addition, the inverse-design process can be extended in a straightforward manner to more general optimization processes, such as topology optimization, by changing the design variables from the current simple geometrical parameters to discrete finite elements^30,31^.

### Experimental realization of a plane-wave generator using the DZIPnC

**Figure 4:**
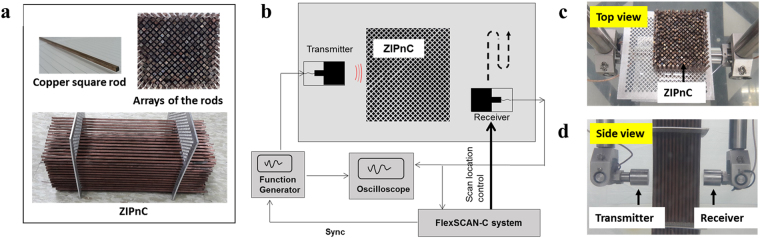
Experimental setup and measurements: (**a)** Copper square rod used for the periodic inclusions, the DZIPnC and corresponding side view. The DZIPnC has a unit-cell lattice of 15 × 13 with *d*_1_ = 2.26 mm and *d*_2_ = 6.34 mm. **(b)** Setup for the experiment on the DZIPnC with the receiver controlled by a motorized scanning system. **(c)** Top-view and **(d)** side-view snapshots of the experiment in a water tank. The transducers on the left and the right are the transmitter and receiver, respectively.

**Figure 5:**
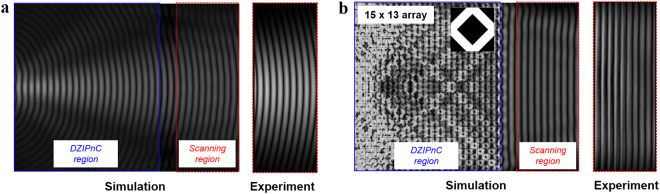
Absolute value of pressure fields simulated and measured: (**a)** without and **(b)** with the DZIPnC. Whereas curved responses are apparent without the crystal, the simulation and experimental results show collimated waves with the DZIPnC.

We experimentally realize the DZIPnC of a Dirac-like point at k = 0 (i.e., the ${\rm{\Gamma }}$ point), particularly designed for underwater ultrasonic applications. Generating plane waves is important in many research and practical fields. For example, in order to assess the capability of a sensor system, testing procedures often require an ideal source such as a plane-wave generator. However, in practice, plane waves are difficult to realize with a single transducer in a limited space, as doing so normally requires a large space satisfying the far-field assumption or an array of single transducers^32^. Using the DZIPnC, plane waves can be generated even with a single transducer within a relatively small space. In this experiment, we designed the DZIPnC following the process shown in Fig. 3. Accordingly, the dimensions in the previous sections are used for the experiment. The DZIPnC was realized with square welding rods of the type readily available for purchase from an online market, and we located them periodically to build the designed arrays of unit cells (Fig. 4a). This shows the simple geometry of the DZIPnC and the easy implementation for practical applications. The experimental setup is shown in Fig. 4b. It is conducted with and without the DZIPnC in a water tank (as in Fig. 4c,d), and two 200 kHz ultrasonic transducers were used as the transmitter and the receiver, scanning an area of 36 × 95 mm^2^ in 0.25 mm steps. The simulation and experimental results are compared in Fig. 5a,b, respectively. It can be clearly observed in Fig. 5b that plane waves are successfully generated with the DZIPnC for in both the numerical simulation and the experiment. In contrast, curved waves are observed without the DZIPnC both theoretically and experimentally, as shown in Fig. 5a. The concept of the proposed DZIPnC-based ultrasonic plane-wave generator can be directly applied to biological imaging applications such as to a nano/micro-particle control system using a plane wave (e.g., acoustic tweezer^33^ and acoustic levitation systems^34^) and can be extended to a wide range of applications by employing an optimal patterning method^35^ (e.g., an ultrasonic omnidirectional wave generator as another ideal source or by means of wave focusing for non-destructive evaluations^36^, biological imaging^37^, and a high-intensity focused ultrasound system^38^).

## Discussion

In conclusion, we have proposed methods which can be used systematically to realize double-zero-index phononic crystals (DZIPnC) with *hard* inclusions. First, the mapping conditions of the DZIPnC are generalized through the spatial symmetry characteristic of Bloch modes. Particularly, it is discovered that phononic crystals with a *hard* inclusion can be mapped to a zero-index medium when its Dirac-like point is at the BZ corner and it originates with a quadrupole mode and two dipole modes. Second, a zone-folding mechanism is introduced which efficiently calculates the effective medium properties *within a unit cell* for *hard*-inclusion phononic crystals, whereas otherwise it is very time-consuming to compute them with arrays of cells. Third, an inverse-design method is suggested using the EMT and bi-objective functions in order to design such crystal structures systematically. Finally, a DZIPnC with a Dirac-like point was designed using the proposed methods and was experimentally realized for the first time. Using arrays of copper welding rods, an underwater plane-wave generator was created. The results from the experimental realization are in good agreement with the numerical simulation results.

## Methods

### Finite element analysis and design optimization

In this paper, the finite element method (FEM) is utilized in the numerical analysis of the DZIPnCs using the commercial software COMSOL Multiphysics. Two types of numerical simulations were conducted: (1) a Bloch-mode analysis with a unit cell, and (2) a time-harmonic analysis with an array of unit cells. First, for the Bloch-mode analysis, a periodic boundary condition based on the Floquet-Bloch theorem is employed at the four boundaries of the unit cell, with mode shapes and band structures then calculated for the unit cells considered in the paper. The triangular meshes are uniformly and symmetrically constructed over the entire domain of the unit cell to be analyzed. Moreover, by using the second order Lagrangian shape function, the Bloch modes are calculated. The calculated Bloch modes show the features of nearly pure dipoles and quadrupoles. Second, a time-harmonic simulation was conducted with the DZIPnC and the effective medium. The largest mesh element size is lower than 1/10 of the shortest wavelength, and perfectly matched layers were used to simulate non-reflective boundaries. The GA toolbox in MATLAB version 2013b is used for the design optimization of the DZIPnC with specified conditions for the target frequency and size of the purchased copper welding rod.

### Measurements and data processing in the experiment

Experiments were conducted in a 1.5 m × 1.3 m × 0.8 m water tank with two motorized arms which scan in the x, y and z directions used for the ultrasonic scanning test. The scanning system is controlled by the FlexSCAN-C system from Sonix. The DZIPnC, made with square-shaped rods, was placed in the water tank. A 200 KHz ultrasonic transducer with a 25 mm diameter (Ultran GS200-D25) was used as a transmitter, positioned 10 mm away from the DZIPnC. An identical transducer is located on the opposite side of the waveguide as a receiver, initially located 10 mm away from the DZIPnC. A function generator (Tektronix AFG3051C) generates 20 sinusoidal waves to the transmitter and the receiver then measures the water movement at every 0.25 mm step, covering an area of 36 × 95 mm^2^ on the *xy* plane. The received signal is digitized by a FlexSCAN-C system with a sampling rate of 100 MHz and a 16-bit resolution. The amplitudes of the received signal were collected at all scanning points at identical time points after receiving ten cycles, after which they were normalized using the maximum value in the data set.

## Electronic supplementary material

Supplementary Note
