# Supplementary material for: Systematic realization of double-zero-index phononic crystals with hard inclusions
Source: Sci Rep. 2018 May 8;8:7288. doi: 10.1038/s41598-018-25696-y (PMC5940914; doi:10.1038/s41598-018-25696-y)
Supplement: Supplementary file 1 — Supplementary Note [file 41598_2018_25696_MOESM1_ESM.docx]

**Supplementary Note for**

**“Systematic realization of double-zero-index phononic crystals with hard inclusions”**

**Jaeyub Hyun^1+^, Wonjae Choi^2,^*, Semyung Wang^1^, Choon-su Park^2^, and Miso Kim^2^**

^1^School of Mechatronics, Gwangju Institute of Science and Technology (GIST),

123 Cheomdan-gwagiro, Buk-gu, Gwangju, 61005, Republic of Korea

^2^Center for Safety Measurement, Korea Research Institute of Standards and Science (KRISS),

267 Gajeong-ro, Yuseong-gu, Daejeon 34113, Republic of Korea

*Corresponding author

Postal Address: Center for Safety Measurement, Korea Research Institute of Standards and Science (KRISS), 267 Gajeong-ro, Yuseong-gu, Daejeon 34113, Republic of Korea.

E-mail Address: [w.choi@kriss.re.kr](mailto:w.choi@kriss.re.kr) Tel. +82-62-868-5749

^+^Present address: Center for Medical Convergence Metrology, Korea Research Institute of Standards and Science (KRISS), 267 Gajeong-ro, Yuseong-gu, Daejeon 34113, Republic of Korea

In the supplementary note, S1) Detailed procedures of boundary field averaging-based homogenization method is provided to numerically compute the effective material properties of the DZIPnC shown in the manuscript. S2) In order to compare with the proposed method, a numerical simulation result of *indirectly* obtaining zero-index characteristics is also described with the original unit cell-based approach. S3) In addition, in order to confirm the versatility of the proposed inverse design method, the DZIPnC with circular inclusions is designed as an example of a different type of the DZIPnC. S4) Moreover, three types of Bloch mode at the Dirac-like point are compared one another. Through this comparison, the effect of a large impedance contrast between hard inclusion and surrounding medium (i.e., water) is clearly investigated. S5) Also, the isotropy of the optimized DZIPnC is verified through IsoFrequency Contour (IFC). S6) Finally, geometrical sensitivity analysis of the DZIPnC according to the changes of the two design variables is conducted to provide a valid range of geometrical dimension that can ensure a zero-index feature.

**S1. Boundary field averaging-based homogenization method.**

In order to design a metamaterial systematically, it is significant to understand the global property of the metamaterial. Homogenization is a process to find its effective material properties and a lot of methods are developed. In this section, we explain a homogenization method based on boundary field averaging. The method has an advantage of no ambiguity problem in sign selection over the conventional scattering parameter-based homogenization method (i.e. S-parameter retrieval method) and, moreover, can be applied to both the time-harmonic analysis and the Bloch mode analysis. This method utilizes both “divergence theorem” and “gradient theorem”, in order to compute boundary field-averaged values on exterior boundaries of a unit-cell or an array of unit-cells. Since it does not require an integration over the entire area, the boundary field averaging-based homogenization method shows the very efficient and robust characteristics in terms of the computational labor. As an example, this homogenization method is applied for extracting the effective material properties of the double negative acoustic metamaterial, and it is performed based on the Bloch mode analysis.

**S1.1. Boundary field averaging-based homogenization.**

Linearized Euler equation in Eq. (S1) and mass balance equation in Eq. (S2) can define relation between pressure (*p*) and velocity fields (***v***). Effective material properties can be obtained through ensemble averaging of the homogenized linearized Euler equation and homogenized mass balance equation, respectively,

$\left\langle\nabla p \right\rangle=j\omega\bar{\boldsymbol{p}}\cdot\left\langle\bar{\boldsymbol{v}} \right\rangle=\left\{ \begin{aligned} j\omega\bar{\rho}_{x}\left\langle v_{x} \right\rangle\\ j\omega\bar{\rho}_{y}\left\langle v_{y} \right\rangle\end{aligned} \right.$ at $\Omega$ (S1)

$\left\langle\nabla\cdot\text{v} \right\rangle=\frac{j\omega\left\langle p \right\rangle}{\bar{\kappa}}$ at $\Omega$ (S2)

where $\left\langle\right\rangle$ is spatial average over the boundary, the bar  $\bar{}$ means ‘effective property of’, and  $\Omega$ is the boundary of region of interest. The effective mass density tensors $\bar{\rho}_{x}$ and $\bar{\rho}_{y}$ and the effective bulk modulus $\bar{\kappa}$ can be obtained from Eq. (S1) and (S2), respectively

$\bar{\rho}_{x}=\frac{\left\langle\nabla p \right\rangle_{x}}{j\omega\left\langle v_{x} \right\rangle}=\frac{1}{j\omega\left\langle v_{x} \right\rangle}\left( \frac{\partial p}{\partial x} \right)$ and $\bar{\rho}_{y}=\frac{\left\langle\nabla p \right\rangle_{y}}{j\omega\left\langle v_{y} \right\rangle}=\frac{1}{j\omega\left\langle v_{y} \right\rangle}\left( \frac{\partial p}{\partial y} \right)$ (S3)

$\bar{\kappa}=\frac{i\omega\left\langle p \right\rangle}{\left\langle\nabla\cdot\boldsymbol{v} \right\rangle}$ (S4)

where $\left\langle\right\rangle_{x}$ is spatial average of *x* direction components over the boundary. Note that, in the manuscript, we use $\bar{\rho}$ for simplicity, rather than $\bar{\rho}_{x}$ and $\bar{\rho}_{y}$, and calculate $\bar{\rho}_{x}$ for $\bar{\rho}$ under the isotropic assumption in which material properties in all direction are identical $\bar{\rho}_{x}=\bar{\rho}_{y}$. The homogenization method based on the boundary field averaging is free from the ambiguity problem basically, since this method integrates directly the local fields of pressure and velocities on the exterior boundaries by using Eq. (S1) and Eq. (S2). In order to solve the equations, we need to obtain the average terms in Eq. (S3) and (S4) on each exterior boundary (i.e., $\left\langle p \right\rangle$, $\left\langle v_{x} \right\rangle$, $\left\langle v_{y} \right\rangle$, $\left\langle\nabla p \right\rangle_{x}$, $\left\langle\nabla p \right\rangle_{y}$, $\left\langle\nabla\cdot\boldsymbol{v} \right\rangle$) which will be described in the next section.

**S1.2. Averaging of the** *p***, *v*,** $\nabla p$**, and** $\nabla\cdot\mathbf{v}$ **on the exterior boundaries.**

Among the averaging terms in the previous section, the averaging of pressure filed *p* and velocity field ***v*** can be obtained straightforwardly, and thus the averaging of them will not be covered here. On the other hand, the averaging process of the other two terms (i.e., $\nabla p$ and$\nabla\cdot\mathbf{v}$) are introduced to compute the averaged values on the boundaries as follows. First, the gradient theorem is utilized in order to compute the averaged value of $\nabla p$. The volume integration can be merely represented by the linear combination of the integrations on exterior boundaries through the gradient theorem as

$\left\langle\nabla p \right\rangle=\frac{1}{a^{2}}\int_{\Omega} \nabla pd\Omega=\frac{1}{a^{2}}\int_{\partial\Omega} p\mathbf{n}\mathrm{dS}$

$=\frac{1}{a^{2}}\left( \int_{\partial\Omega_{L}} -pdS+\int_{\partial\Omega_{R}} pdS+\int_{\partial\Omega_{B}} -pdS+\int_{\partial\Omega_{T}} pdS \right)$ (S5)

where $\Omega$ with the subscripts *L, R, B* and *T* mean left, right, bottom, and top boundaries, respectively.

Next, the divergence theorem is utilized in order to compute the averaged value of $\nabla\cdot\mathbf{v}$. Here the particle velocity vector (**v**) can be easily obtained from the linearized Euler equation.

$\left\langle\nabla\cdot\mathbf{v} \right\rangle=\frac{1}{a^{2}}\int_{\Omega} \nabla\cdot\mathbf{v}d\Omega=\frac{1}{a^{2}}\int_{\partial\Omega} \boldsymbol{n\cdot v}\mathrm{dS}$

$=\frac{1}{a^{2}}\left( \int_{\partial\Omega_{L}} -v_{x}dS+\int_{\partial\Omega_{R}} v_{x}dS+\int_{\partial\Omega_{B}} -v_{y}dS+\int_{\partial\Omega_{T}} v_{y}dS \right)$ (S6)

where $v_{x}=\frac{1}{j\omega\rho}\frac{\partial p}{\partial x}$ and $v_{y}=\frac{1}{j\omega\rho}\frac{\partial p}{\partial y}$

**S1.3. Example: DZIPnC with circular inclusions.**

In order to verify the robustness and performance of the boundary field averaging-based homogenization method, we consider an acoustic metamaterial which exhibits the near-zero effective mass density and the near-infinite effective bulk modulus. Compared with the conventional S-parameter retrieval method, the boundary field averaging-based method has the advantage that it can conduct the homogenization not only based on Bloch mode analysis, but also based on time-harmonic analysis. The double-zero-index phononic crystal (DZIPnC) is designed through the rubber cylinder in water matrix as shown in Fig. S1. The material properties used are as follows: $\rho_{water}=1000 kg/m^{3}$ and $c_{water}=1500 m/s$ for water and $\rho_{rubber}=1300 kg/m^{3}$and $c_{rubber}=460 m/s$ for rubber, and the operational frequency is about 38,100 Hz.

**
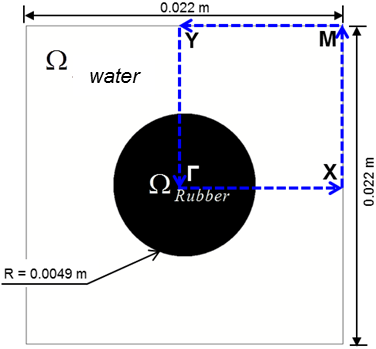
**

Figure S1. Unit cell configuration of the considered DZIPnC.

Band structure of the DZIPnC is in Fig. S2a. It has the Dirac-like point at about 38,100 Hz. The homogenization of the DZIPnC is conducted in order to observe whether the characteristic of near zero-index really occurs at about 38,100 Hz. Table S1 shows the effective material properties of the DZIPnC extracted by the boundary field averaging-based homogenization from Eq. (S3) and (S4). Here, since the mass density of water is 1000 kg/m^3^, the real part of effective material properties normalized by the properties of water has near zero values as represented in Table S1. Fig. S2b shows the comparison of IsoFrequency Contour (IFC) between the actual DZIPnC and the homogenized DZIPnC with the effective material properties, and the two IFCs are in a good agreement with each other. The IFC with the effective material properties can be plotted by using the equation represented in Fig. S2b. We can see from the results that the boundary field averaging-based homogenization method works well with Bloch mode analysis.

Table S1. The effective material properties of the DZIPnC based on the Bloch mode analysis.

| Effective material properties | Real part | Imaginary part |
| --- | --- | --- |
| Effective mass density (kg/m^3^) | 90.0 | 11.8 |
| Effective bulk modulus (Pa) | 1.7e+11 | 7.9e+9 |

**
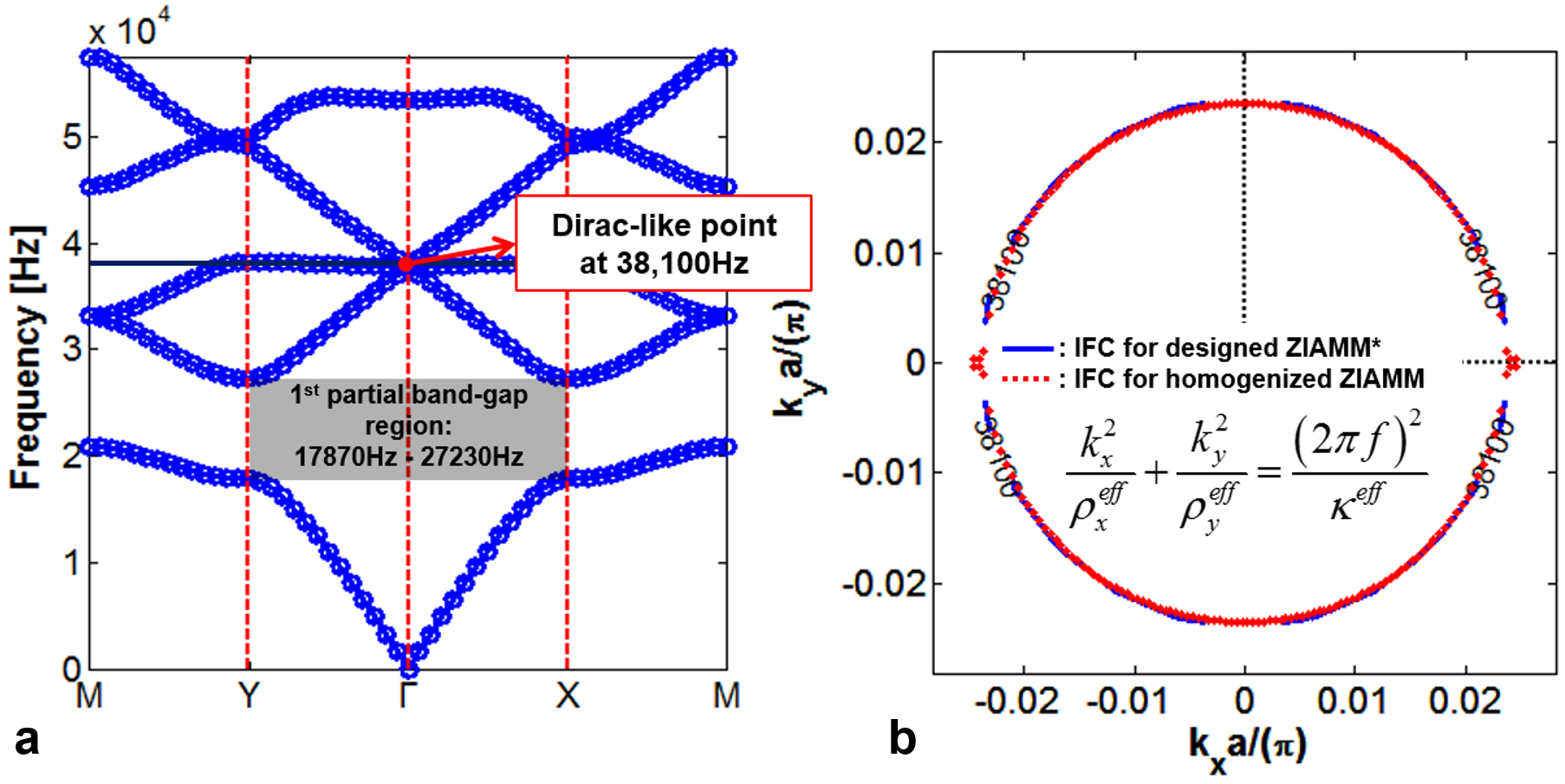
**

Figure S2. Homogenization based on the Bloch mode analysis. (a) Band structure of the DZIPnC (b) Comparison between IFCs from the numerical simulation with the unit cell and from the effective material properties.

The DZIPnC can be applied to the acoustic tunneling system for the lossless transmission of acoustic energy. The almost 100% lossless transmission of acoustic energy is possible through the waveguide composed of the DZIPnC as shown in Fig. S3b. Moreover, the effective material properties of the DZIPnC computed by the homogenization method show the similar lossless transmission result, as represented in Fig. S3c. Also, the DZIPnC is applied to the self-collimator system for the acoustic wavefront control. The refractive index of the DZIPnC is near zero. The angle of the transmitted acoustic wave is near zero from the Snell’s law. Thus transmitted acoustic wave always propagates along the normal direction to the surface of waveguide, regardless of the incident wave direction. The acoustic wavefront can be controlled by varying the shape of waveguide. To confirm this concept of the wavefront control, DZIPnC -based acoustic self-collimator system is presented. The waveguide composed of the DZIPnC can transform the cylindrical wavefront of a monopole source into the plane wavefront, as shown in Fig. S4b. Then, in order to verify the homogenization method, we compare the results between the effective medium with the homogenized material properties of the DZIPnC and the actual DZIPnC. As represented in Fig. S4c, the result with effective material properties is almost identical to result in Fig. S4b.


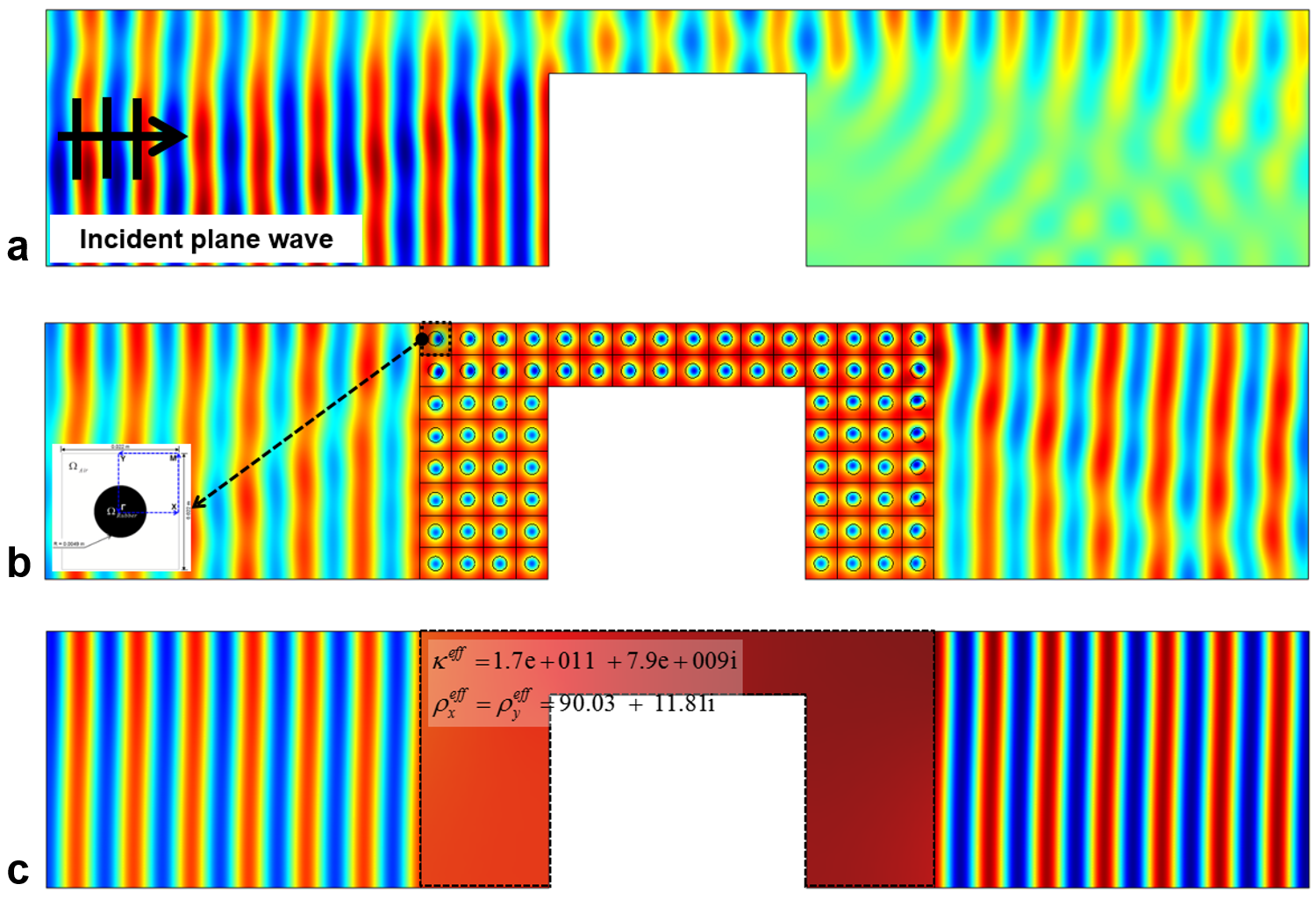


Figure S3. Finite element simulations showing the acoustic tunneling effect using DZIPnC. (a) Acoustic pressure through the narrow channel without the DZIPnC (b) acoustic pressure through the narrow channel with the DZIPnC (c) acoustic pressure through the narrow channel with the effective material properties of the DZIPnC.


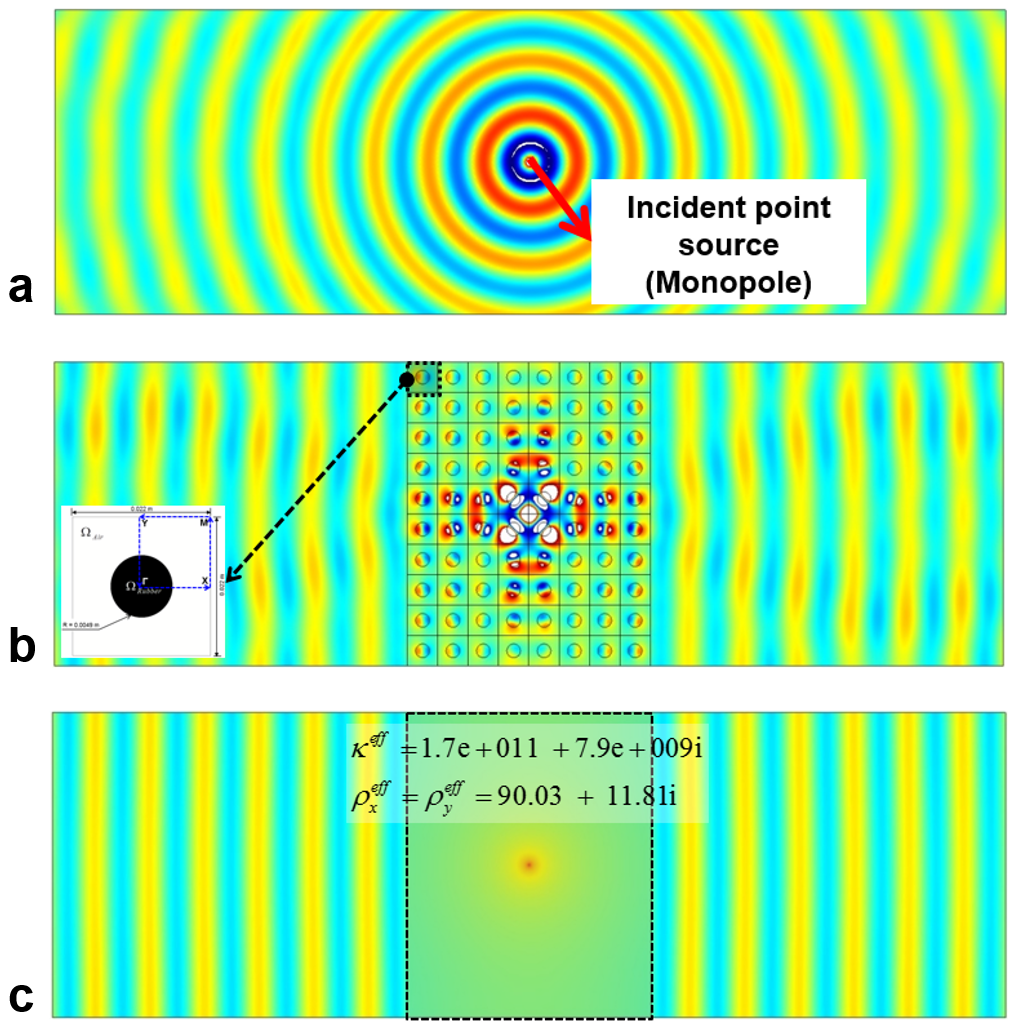


Figure S4. Finite element simulations of acoustic wavefront control (i.e., self-collimating) using the DZIPnC. (a) Acoustic pressure of a monopole source without the DZIPnC (b) acoustic pressure of a monopole source with the DZIPnC (c) acoustic pressure of a monopole source with the effective material properties of the DZIPnC.S2. Zero-index characteristics obtained “*indirectly*” with the original unit cell.

In the manuscript, we homogenize a unit cell to have effective material properties. In such manner, we can calculate the refractive index straightaway with the properties. However, for some unit cells such as the original unit cell in the manuscript, it is impossible to compute the effective properties *directly* through the homogenization. One way to verify the characteristics of the metamaterial is to conduct a simulation with an array of unit cells, every time the cell shape is updated, which we call *indirect* estimation. One example is shown in Fig. S5. From the result of the harmonic analysis as in Fig. S5a, the zero-index characteristic is calculated by the S-matrix method or homogenization method over the entire array boundary, not unit cell boundary. Fig. S5a shows the simulation for the final update stage in the DZIPnC optimization process, showing no phase distortion or infinite phase speed within the crystal and thus zero refractive index. The DZIPnC can then show to control the shape of acoustic wavefront regardless of the directivity of input source, as shown in Fig. S5b. Thus, it is possible to use the *indirect* estimation for a design process, but it is computationally more expensive than the *direct* estimation we proposed in the manuscript.


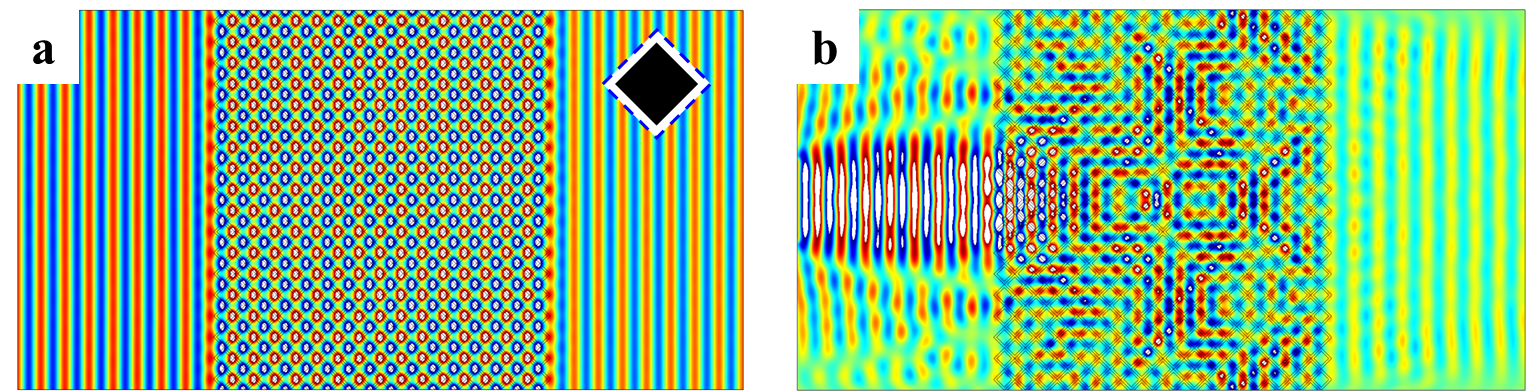


Figure S5. Finite-element time-harmonic simulation with an array of the original unit cells for *indirect* estimation of zero-index characteristics. (a) with a plane wave incidence showing no phase delay (b) with a finite-size wave incidence.

**S3. Inverse design of the DZIPnC with the circular inclusion.**

In this section, we describe another example of DZIPnC to show our homogenization and optimization method are applicable to any crystal shape. A design optimization is carried out for a DZIPnC with a circular-type inclusion. Inside Fig. S6 shows configuration of the DZIPnC optimized by the method proposed in the manuscript, and the size of unit cell and the inclusion diameter are 26.2 mm and 10.5 mm respectively. The optimized DZIPnC is SUS308 inclusion ($\rho_{SUS308}=8,000 kg/m^{3}$ and$c_{SUS308}=5,772 m/s$) in water background. As shown in Fig. S6 and Table S2, the optimized DZIPnC with the circular inclusion has a size of approximately 26.2 mm for the target operational frequency, 50 kHz. The zero-index characteristic can be confirmed by the numerical analysis of an array of the optimized DZIPnC with the circular inclusion, as represented in Fig. S6. Therefore, we can confirm that the proposed inverse design approach can be applied to the various types of square crystals with $C_{4\nu}$ symmetry.


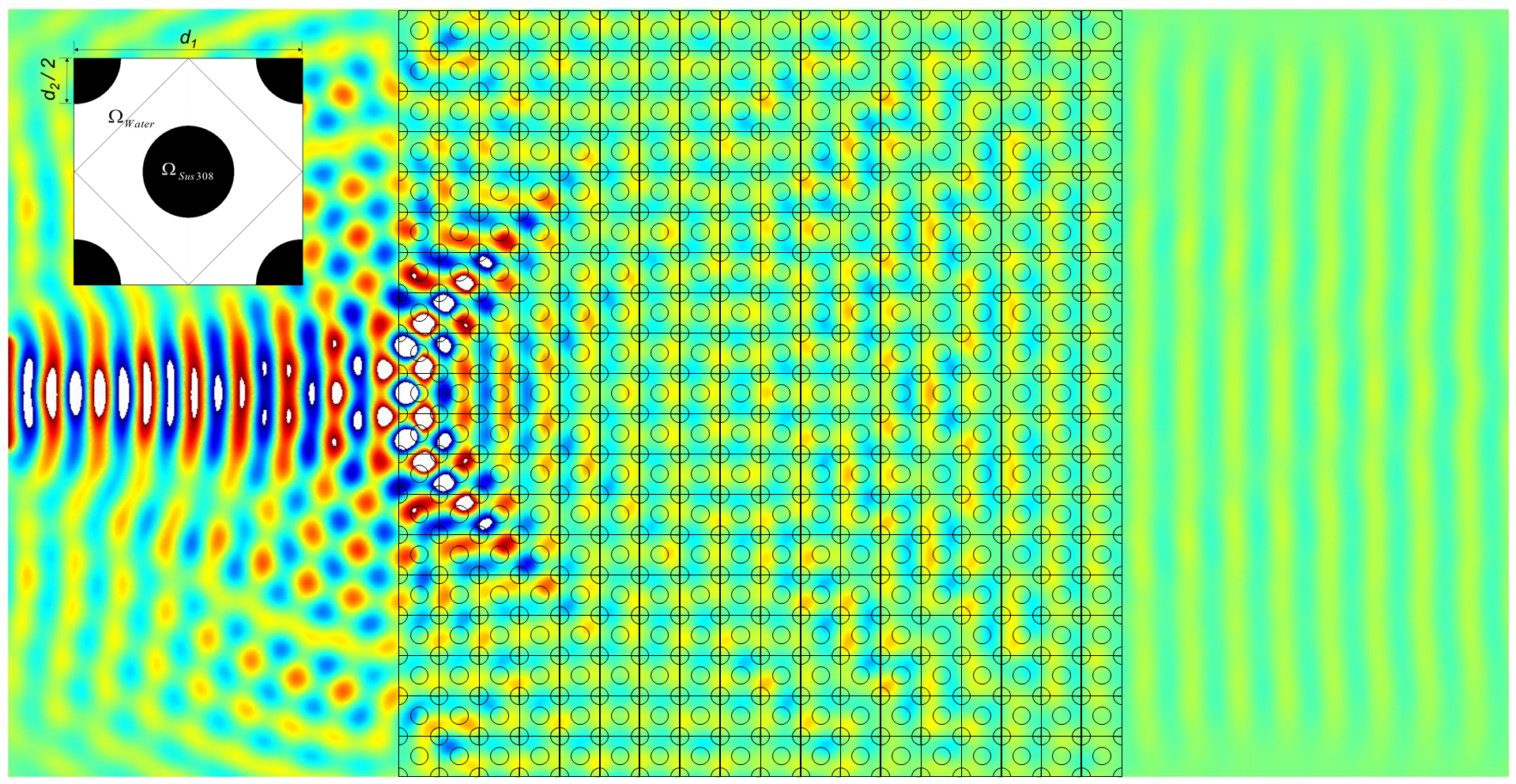


**Figure S6. Finite element simulation for the optimized DZIPnC with the circular inclusion designed by the proposed inverse design method.** The wave transmission presents plane wave generation (inside: The configuration of optimized DZIPnC with circular inclusion)

Table S2. The dimension of the optimized DZIPnC with the circular inclusion.

| Target operational frequency (kHz) | d_1_: Size of unit cell (mm) | d_2_: Diameter of inclusion (mm) |
| --- | --- | --- |
| 50 | 26.2 | 10.5 |

**S4. The effect of a large impedance contrast between hard inclusion and surrounding medium.**

It is important to note that a physically meaningful comparison of the transmission performance of the acoustic wave can be conducted by means of the acoustic intensity (i.e., energy) expressed as the product of acoustic particle velocity and pressure, instead of the acoustic pressure. In order to show this fact more clearly, as the first step, both transmission ($\boldsymbol{T}_{\boldsymbol{p}}$) and reflection ($\boldsymbol{R}_{\boldsymbol{p}}$) coefficients of acoustic pressure are should be considered on the interface between two different media. These can be expressed as following Eq. (S7) [1]. Here, the normal incidence of incident angle 0 can be considered without loss of generality.

$\boldsymbol{R}_{\boldsymbol{p}}\boldsymbol{=}\frac{\boldsymbol{P}_{\boldsymbol{r}}}{\boldsymbol{P}_{\boldsymbol{i}}}\mathbf{=}\frac{\boldsymbol{Z}_{\boldsymbol{1}}\boldsymbol{-}\boldsymbol{Z}_{\boldsymbol{0}}}{\boldsymbol{Z}_{\boldsymbol{1}}\boldsymbol{+}\boldsymbol{Z}_{\boldsymbol{0}}}$ (S7a)

$\boldsymbol{T}_{\boldsymbol{p}}\boldsymbol{=}\frac{\boldsymbol{P}_{\boldsymbol{t}}}{\boldsymbol{P}_{\boldsymbol{i}}}\mathbf{=}\frac{\boldsymbol{2}\boldsymbol{Z}_{\boldsymbol{1}}}{\boldsymbol{Z}_{\boldsymbol{1}}\boldsymbol{+}\boldsymbol{Z}_{\boldsymbol{0}}}$ (S7b)

where $\boldsymbol{Z}_{\boldsymbol{0}}$ is the characteristic impedance of incident medium, and $\boldsymbol{Z}_{\boldsymbol{1}}$ is the characteristic impedance of transmitted medium. As shown in this equation, the transmission and reflection coefficients can be easily expressed by the characteristic impedance of the two media. Next, let us take into account the unit cell of the DZIPnC covered in our study by using these equations. In this case that the transmitted medium (i.e., hard inclusion) has an extremely large impedance compared to the incident medium (i.e., $\boldsymbol{Z}_{\boldsymbol{1}}\boldsymbol{\gg}\boldsymbol{Z}_{\boldsymbol{0}}$), the transmitted pressure ($\boldsymbol{P}_{\boldsymbol{t}}$) is about twice as large as the incident pressure ($\boldsymbol{P}_{\boldsymbol{i}}$). We may think that this result is a confusing part of you. However, the case of acoustic particle velocity shows completely different phenomenon. The transmission ($\boldsymbol{T}_{\boldsymbol{v}}$) and reflection ($\boldsymbol{R}_{\boldsymbol{v}}$) coefficients of acoustic particle velocity can be represented by Eq. (S8).

$\boldsymbol{R}_{\boldsymbol{v}}\boldsymbol{=}\frac{\boldsymbol{V}_{\boldsymbol{r}}}{\boldsymbol{V}_{\boldsymbol{i}}}\mathbf{=}\frac{\boldsymbol{Z}_{\boldsymbol{1}}\boldsymbol{-}\boldsymbol{Z}_{\boldsymbol{0}}}{\boldsymbol{Z}_{\boldsymbol{1}}\boldsymbol{+}\boldsymbol{Z}_{\boldsymbol{0}}}$ (S8a)

$\boldsymbol{T}_{\boldsymbol{v}}\boldsymbol{=}\frac{\boldsymbol{V}_{\boldsymbol{t}}}{\boldsymbol{V}_{\boldsymbol{i}}}\mathbf{=}\frac{\boldsymbol{2}\boldsymbol{Z}_{\boldsymbol{0}}}{\boldsymbol{Z}_{\boldsymbol{1}}\boldsymbol{+}\boldsymbol{Z}_{\boldsymbol{0}}}$ (S8b)

As shown in the equation (Eq. S8b) for the transmission coefficient of acoustic particle velocity, the transmitted velocity ($\boldsymbol{V}_{\boldsymbol{t}}$) is near zero within the hard inclusion with a large impedance because the characteristic impedance of transmitted medium is even larger than that of incident medium (i.e.,$\boldsymbol{Z}_{\boldsymbol{1}}\boldsymbol{\gg}\boldsymbol{Z}_{\boldsymbol{0}}$). Consequently, the acoustic intensity expressed as the product of acoustic particle velocity and pressure also has near zero, which is negligibly small. This fact shows that the assumption (i.e., copper or iron can be regarded as rigid material compared to the surrounding medium due to the large impedance contrast) used in our study is valid.

In order to verify that this assumption can be equally applied to the optimized DZIPnC presented in this study, Bloch mode analysis for the unit cell of the optimized DZIPnC was performed through COMSOL Multiphysics. The results of Bloch mode analysis are represented well in Fig. S7. Figure S7a shows both the band structure and the Dirac-like point for the unit cell of the optimized DZIPnC. Moreover, Fig. S7b presents the three types of Bloch modes generated at the Dirac-like point (i.e., two dipoles and one quadrupole), where the upper panel shows the acoustic pressure, the middle panel shows the acoustic particle velocity, and the lower panel shows the acoustic intensity. Here, the acoustic particle velocity is calculated using the conventional Euler’s equation (i.e.,$\boldsymbol{v}=\left( {-1}/{j\omega\rho} \right)\nabla p$). As expected, both acoustic particle velocity and intensity are near zero within the hard inclusion with a large impedance. In conclusion, it is physically reasonable to compare the transmission and reflection performances by using acoustic intensity. Nevertheless, in general, acoustic pressure is presented in many studies because it most clearly show the shape of the Bloch modes excited within the unit cell [2-4].


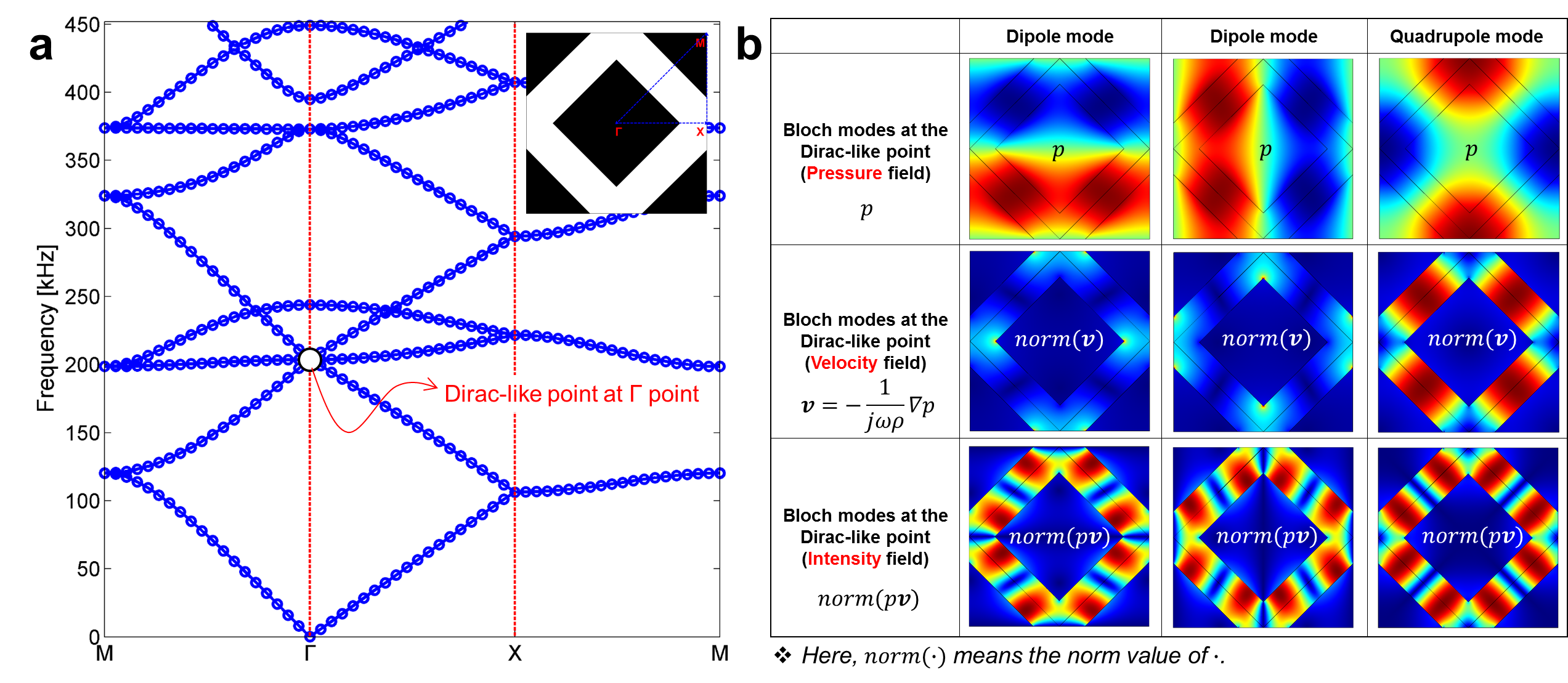


**Figure S7. Characteristics of the unit cell of the optimized DZIPnC. (a)** Band structure of the optimized DZIPnC. The DZIPnC has the Dirac-like point at $\Gamma$ point. **(b)** Bloch modes at the Dirac-like point, upper panel: acoustic pressure field, middle panel: acoustic particle velocity field, and lower panel: acoustic intensity field.

**S5. Characterization of isotropy of the optimized DZIPnC through IsoFrequency Contour (IFC).**

To characterize the anisotropy or isotropy of the optimized DZIPnC, we performed the Bloch mode analysis, thereby calculating the band structure (Fig. S8a) and obtaining the IsoFrequency Contour (IFC) of the 2^nd^ band in **k**-space (Fig. S8b). Here, IFC is nearly perfect circular within the finite region where the Dirac-like point occurs and the wavenumber (**k**) is close to zero (i.e., near $\Gamma$ point), as shown in Fig. S8c. In other words, this means that the optimized DZIPnC can fully guarantee the isotropic characteristic in this finite region. However, since a unit cell with a square-shaped inclusion constructs a basic square lattice, anisotropy is increased in the high-wavenumber region. The anisotropy in the high-wavenumber region can be confirmed by the noncircular shape of IFC in Fig. S8b. Of course, in order to take advantage of this anisotropy, several waveguide systems, such as self-collimator and angular filters, have been extensively studied. To sum up, the isotropy of the optimized DZIPnC can be guaranteed at the finite operational frequency range at which the Dirac-like point is generated. Meanwhile, the isotropy of the optimized DZIPnC somehow contradicts a common understanding that the dispersion relation of the phononic crystal in a square lattice is in general anisotropic. In fact, this special characteristic (i.e., isotropy) is a result of accidental triple degeneracy caused by intersection between linear bands and a flat band at the Dirac-like point [2].


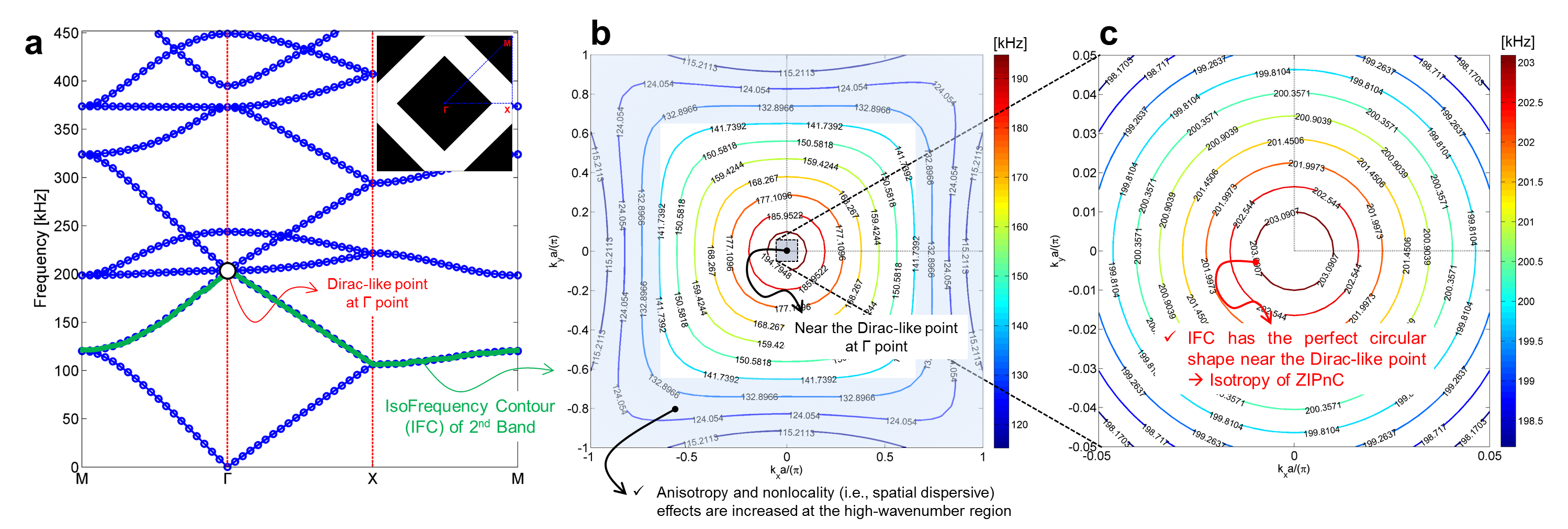


**Figure S8.** **Characterization of isotropy of the optimized DZIPnC through IsoFrequency Contour (IFC) analysis.** **(a)** Band structure of the optimized DZIPnC. **(b)** IFC of the 2^nd^ band in the entire irreducible Brillouin zone. **(c)** IFC of the 2^nd^ band near the zone center (i.e., $\Gamma$ point) representing the Dirac-like point.

**S6. Geometrical sensitivity analysis of the DZIPnC according to the changes of the two design variables.**

Geometrical sensitivity on the performance is a very important point from an engineering point of view. We conducted a geometrical sensitivity analysis according to the changes of $d_{1}$ and$d_{2}$. Through this sensitivity analysis, a valid range of geometrical dimension that can ensure a zero-index feature is provided.

First, we changed only $d_{2}$ by $\pm1$0 % (Fig. 1), and the operational frequency were shifted by $\mp$20 kHz, respectively, which is expected since $d_{2}$ is the lattice constant of unit cell. In order to focus on the cases with a fixed operation frequency, we decided to perform the sensitivity analysis by changing only $d_{1}$ with fixed $d_{2}$ in the next paragraph.


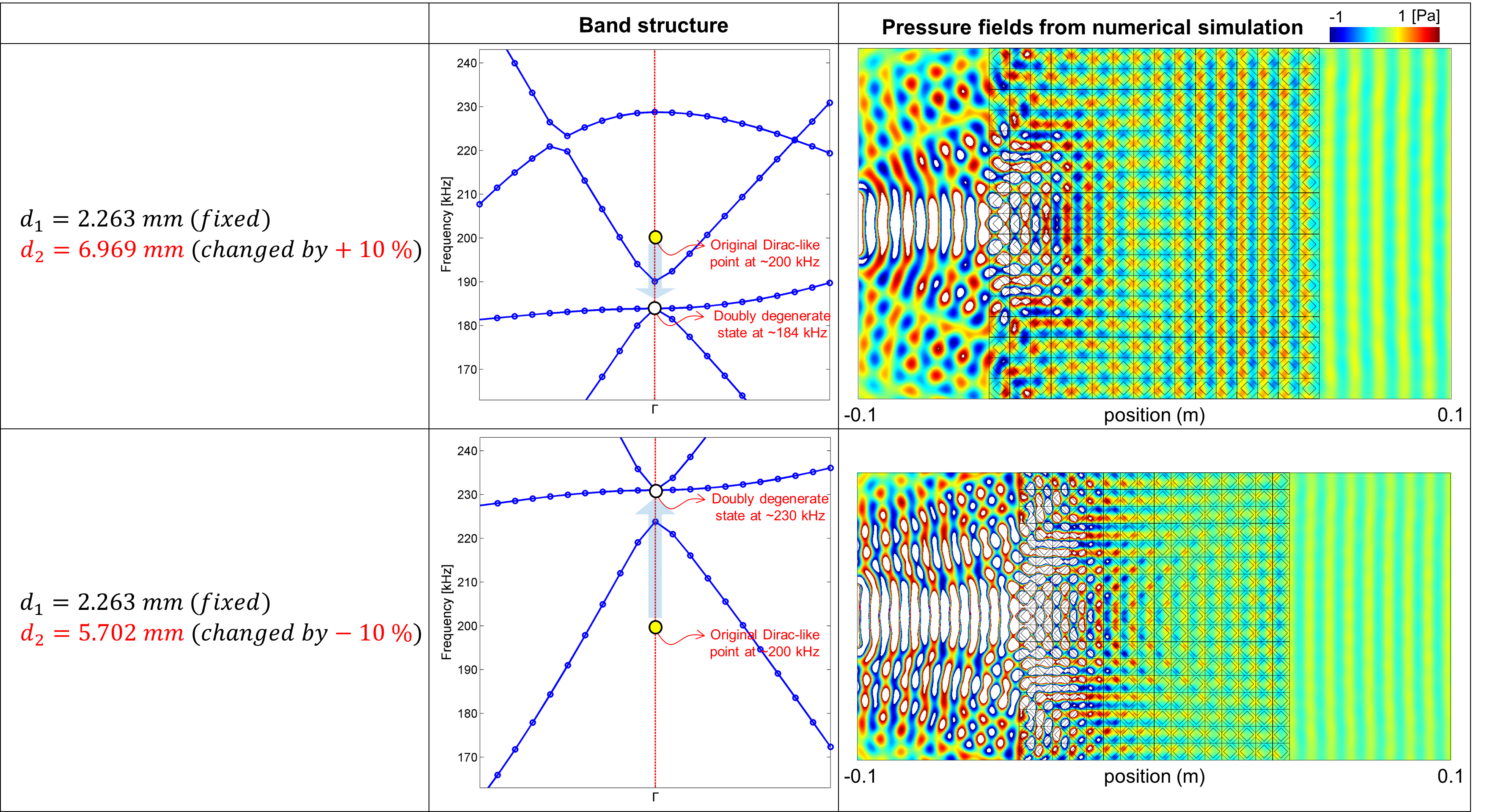


**Figure S9. Geometrical sensitivity of the optimized DZIPnC on the zero-index feature as the** $\boldsymbol{d}_{\boldsymbol{2}}$ **changes.** The $d_{2}$ is increased and decreased by 10 %, respectively. Changing the lattice constant of the unit cell ($d_{2}$) alters the target operational frequency.

Second, we also carried out the sensitivity analysis by changing $d_{1}$, shown in Fig. 2 and 3. As $d_{1}$ decreases by -5% to -10%, the lower two bands among the three constructing the Dirac-like point maintain almost the same target operational frequency at approximately 200 kHz, and only the top band is shifted to a higher frequency as in Fig. 2. We performed the sensitivity analysis with increasing $d_{1}$ by +5%, as shown in Fig. 3. Contrary to the case of decreasing $d_{1}$, only the bottom one of the three bands maintains almost the identical target operational frequency (i.e., ~200 kHz), and the other two bands are shifted to a higher frequency. In the cases considered in the analysis, all simulation results show plane waves. In summary, from the sensitivity analyses according to$d_{1}$, we can confirm that the near-zero-index feature (i.e., minimizing a phase change through the waveguide) is guaranteed in the range of about $\pm$10 %. In this range, the DZIPnC exhibits a single zero-index feature due to double degenerate states. Therefore, as shown in Figs. 2 and 3, the acoustic pressure of transmitted wave is greatly reduced because of a large impedance mismatch between the DZIPnC with a single zero-index feature and the surrounding medium (i.e., water). However, this impedance mismatch problem may not be a critical issue in systems where amplification of input power can be guaranteed.


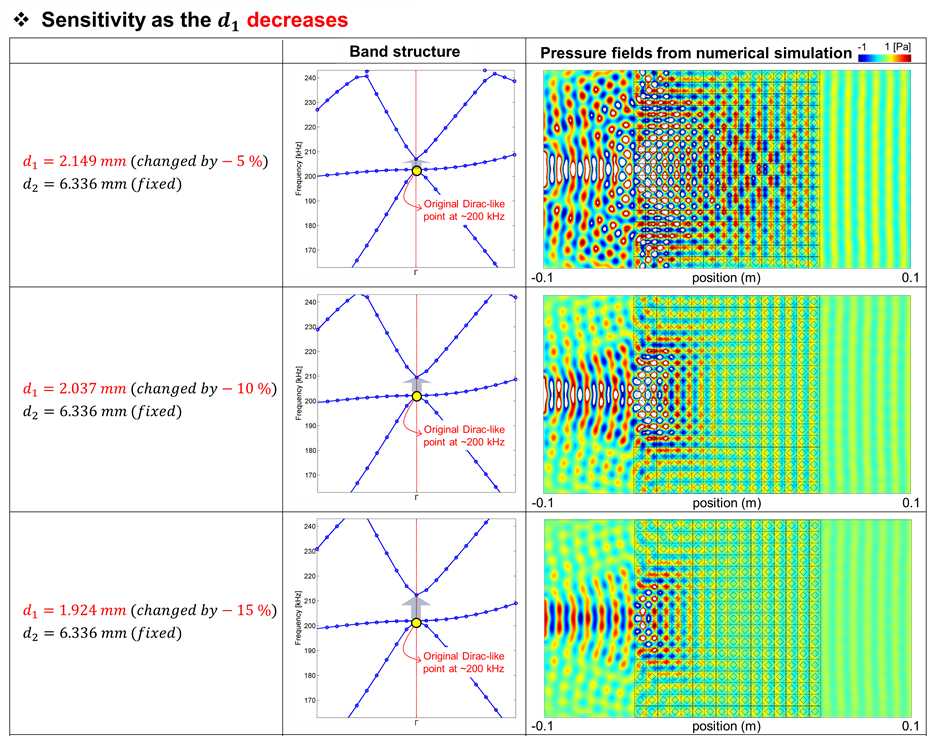


**Figure S10. Geometrical sensitivity of the optimized DZIPnC on the zero-index feature as the** $\boldsymbol{d}_{\boldsymbol{1}}$ **decreases.**


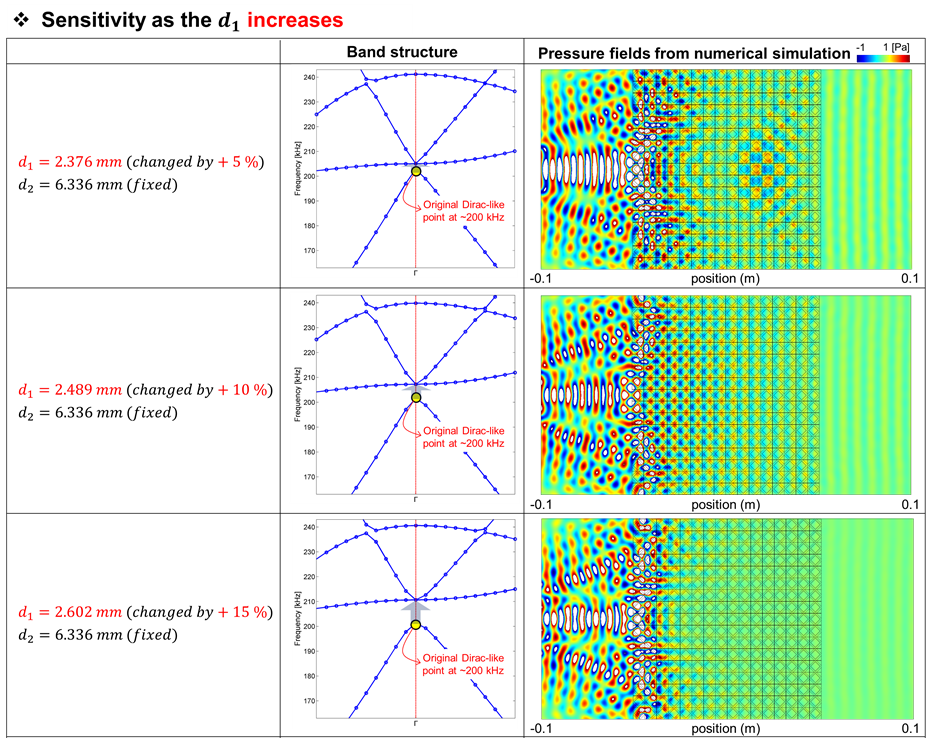


**Figure S11. Geometrical sensitivity of the optimized DZIPnC on the zero-index feature as the** $\boldsymbol{d}_{\boldsymbol{1}}$ **increases.**

**References**

| [1] | L. E. Kinsler, A. R. Frey, A. B. Coppens & J. V. Sanders, "Fundamentals of acoustics", 222 Rosewood Drive, Danvers, Ma, 01923: *John-Wiely & Sonc, Inc*, (2000). |
| --- | --- |
| [2] | Z.-G. Chen *et. al.*, "Accidental degeneracy of double Dirac cones in a phononic crystal," *Sci. Rep*. **4**, 4613, (2014). |
| [3] | L. Y. Zheng *et. al.*, "Acoustic cloaking by a near-zero-index phononic crystal," *Appl. Phys. Lett.,* **104**, 161904, (2014). |
| [4] | Y. Li, Y. Wu & J. Mei, "Double Dirac cones in phononic crystals," *Appl. Phys. Lett.,***105**, 014107, (2014). |
